# Correlation analysis of differentially expressed long non-coding RNA HOTAIR with PTEN/PI3K/AKT pathway and inflammation in patients with osteoarthritis and the effect of baicalin intervention

**DOI:** 10.1186/s13018-023-03505-1

**Published:** 2023-01-12

**Authors:** Xiaolu Chen, Jian Liu, Yanqiu Sun, Jianting Wen, Qin Zhou, Xiang Ding, Xianheng Zhang

**Affiliations:** 1 Anhui University of Traditional Chinese Medicine, Hefei, 230031 Anhui Province China; 2 Department of Rheumatology and Immunology, First Affiliated Hospital of Anhui University of Traditional Chinese Medicine, Hefei, 230038 Anhui Province China; 3 Institute of Rheumatology, Anhui University of Chinese Medicine, Hefei, 230012 Anhui Province China

**Keywords:** Osteoarthritis, Long non-coding RNA HOTAIR, Baicalin, Osteoarthritis chondrocytes, PTEN/PI3K/AKT pathway

## Abstract

**Objective:**

This study aims to investigate the correlation of long non-coding RNA HOX transcript antisense RNA (lncRNA HOTAIR) with the PTEN/PI3K/AKT pathway and clinical-related indicators in osteoarthritis (OA) and determine the effect of baicalin intervention.

**Methods:**

The levels of clinical lipid metabolism indexes and immune-inflammatory indexes in OA patients and normal controls was detected. OA chondrocytes (OA-CHs) were induced with peripheral blood mononuclear cells (PBMCs), followed by baicalin treatment (50 ug/mL). RT-qPCR was performed to measure lncRNA HOTAIR expression. The levels of inflammatory cytokines and adiponectin were detected using ELISA kits. CCK-8 assay was used to assess the viability of CHs. The related protein expression was measured using Western blot analysis.

**Results:**

LncRNA HOTAIR might act as a biomarker of OA in vivo. LncRNA HOTAIR was positively correlated with TC, hs-CRP, IgA, TNF-α, and VAS score. Overexpression of lncRNA HOTAIR in vitro inhibited cell proliferation, reduced IL-10 and PTEN expression, but augmented TNF-α, p-PI3K, and p-AKT proteins in OA-CHs stimulated by OA-PBMCs. The changes of above indexes were also observed in OA-CHs stimulated by OA-PBMCs treated with si-lncRNA HOTAIR or baicalin, implying the synergistic effects of baicalin and lncRNA HOTAIR silencing on OA.

**Conclusions:**

Conclusively, lncRNA HOTAIR was highly expressed in OA-CHs, which facilitated OA inflammatory responses by orchestrating inflammatory cytokines and the PTEN/PI3K/AKT pathway. Baicalin exerted therapeutic effects by inhibiting the expression of lncRNA HOTAIR, decreasing the protein levels of p-PI3K and p-AKT, and increasing the protein levels of PTEN, APN, and ADIPOR1.

## Introduction

Osteoarthritis (OA) is a chronic degenerative joint disease featured by low-level inflammation and cartilage degeneration [1, 2], leading to irreversible structural and functional changes in the joint. Clinical signs associated with OA include joint pain, swelling, and significant functional impairment. Multiple risk factors for OA have been reported, including age, joint trauma, obesity, and genetic susceptibility [3]. To a certain extent, obesity is accompanied with a state of chronic inflammation. Hyperlipidemia is a common metabolic change characterized by an abnormal increase in serum total cholesterol (TC), triglycerides, and low-density lipoprotein cholesterol, as well as an abnormal decrease in high-density lipoprotein cholesterol and apolipoprotein A1, all of which contribute to the inflammatory response and OA deterioration. In addition, body fat, insulin resistance index, and body mass index are negatively related to adiponectin (APN) [4, 5]. APN, a recently discovered cytokine released by adipose tissue, is a regulator for inflammatory resolution in OA [6]. Emerging evidence [7] has demonstrated the involvement of long non-coding RNAs (lncRNAs) in the inflammation, lipid metabolism, and other processes of OA, serving a fundamental role in the development and progression of OA [8].

A prior research [9] has observed that lncRNA HOX transcript antisense RNA (HOTAIR) expression in OA-chondrocytes (CHs) was apparently higher than that in normal CHs, and OA-CHs transfected with small interfering RNA (si)-lncRNA HOTAIR showed a stronger proliferation capacity than those transfected with si-negative control (NC). The tumor suppressor gene phosphatase and tensin homolog deleted on chromosome ten (PTEN) can dephosphorylate and inactivate phosphatidylinositol-3,4,5-trisphosphate (PIP3), an important molecule in the phosphoinositide 3-kinase (PI3K)/AKT pathway, to downregulate the PI3K/AKT pathway. The PTEN/PI3K/AKT pathway is considered a classic inflammatory pathway. PTEN can downregulate the expression of fatty acid synthase by inhibiting the PTEN/PI3K/AKT pathway [10]. Interleukin (IL)-10 and tumor necrosis factor-α (TNF-α) are the critical pro-inflammatory and anti-inflammatory factors of OA [11], respectively. Of note, the PTEN/PI3K/AKT pathway can increase TNF-α expression and decrease IL-10 expression [12]. Furthermore, lncRNA HOTAIR silencing can obviously reduce the content of TNF-α and enhance the content of IL-10 [13]. However, whether lncRNA HOTAIR can assume a role in immune regulation by affecting the PTEN/PI3K/AKT pathway in the inflammatory response of OA has not been clearly clarified yet.

Baicalin, an important flavonoid isolated from the dried roots of Scutellaria Baicalensis Georgi, is a powder of light-yellow color with bitter taste at room temperature. Baicalin possesses diverse pharmacological properties such as anti-inflammation, cholesterol lowering, and anti-allergic. Importantly, the effectiveness of baicalin in controlling OA progress has also attracted considerable concern. For example, it has been reported that treatment with baicalin not only prevents cartilage destruction but also alleviates IL-1β-induced inflammatory injury and represses the production of IL-6 and TNF-α in OA [14, 15]. These findings suggest that baicalin may be a potential agent in the treatment of OA. However, the exact mechanism of baicalin in OA is still unclear.

Therefore, this study focused on the expression pattern lncRNA HOTAIR in OA patients and analyzed its correlation with clinical indicators and visual analog scale (VAS) scores. Finally, lncRNA HOTAIR expression was intervened in cell experiments to assess its regulatory effect on the PTEN/PI3K/AKT pathway, inflammatory factors, and lipid metabolism indexes. Moreover, the involvement of baicalin in the development of immune inflammatory response in OA-CHs was determined, and the synergistic effect between baicalin and HOTAIR in the treatment of osteoarthritis was explored.

## Materials and methods

### Subjects and samples

OA patients and normal control (NC) subjects matched on gender and age were enrolled from the First Affiliated Hospital of Anhui University of Traditional Chinese Medicine between November 2020 and May 2021. Patients who did not meet the following criteria were excluded: not conforming to the 2019 Guidelines for the Diagnosis of OA [16], severe mental illness, significant liver or renal function impairment, the administration of immunosuppressive drugs, or pregnancy. This study was conducted following the relevant provisions of the *Declaration of Helsinki* [17] and was approved by the Ethical Committee of Scientific Research of Anhui University of Traditional Chinese Medicine's First Affiliated Hospital [2014AH-06(J)].

### Co-culture of peripheral blood mononuclear cells (PBMCs) with OA-CHs and cell transfection

Totally, 5 mL venous blood was collected from normal healthy subjects and OA patients, and diluted with an equal volume of normal saline. The same amount of lymphocyte separation solution was slowly added into the prepared samples and centrifuged at 2000 r/min for 20 min. The white floc in the centrifuge tube was transferred into a clean centrifuge tube. The same amount of normal saline was added into the tube and mixed, followed by centrifugation at 800 r/min for 8 min and rinsing twice. Afterward, the samples were transferred into a culture flask and incubated for later use.

OA-CHs and baicalin were purchased from Saibaikang Biotechnology Co., Ltd. (Shanghai, China). PBMCs were seeded into the Transwell chamber and cultured in 75 µL Dulbecco’s Modified Eagle Medium (DMEM). CHs were digested and centrifuged. Then, the medium was discarded, and CHs were washed twice with phosphate buffer saline, resuspended with medium, and seeded into the basolateral chamber, followed by the supplementation of 100 µL DMEM. Following cell adherence, different proportions of OA-PBMCs were added into the apical chamber. Finally, the optimal stimulation concentration of PBMCs was screened by cell counting kit-8 (CCK-8) assay. On the basis of PBMC stimulation concentration (3:1, 48 h), the above steps were repeated, and baicalin at different concentrations was added into the basolateral chamber to screen the optimal concentration of baicalin. Co-culture was conducted in DMEM containing 100 U/mL penicillin and 0.1 mg/mL streptomycin with 5% CO_2_ at 37 °C until the confluence of CHs reached 70–90%. OA-CHs were transfected with PCDNA3.1-lncRNA HOTAIR plasmid, si-lncRNA HOTAIR plasmid, and corresponding NC plasmids using Lipofectamine 2000, respectively. After 24 h, the cells were harvested for subsequent analysis. All plasmids were purchased from GenePharma (Shanghai, China).

### Reverse transcription-quantitative polymerase chain reaction (RT-qPCR)

The total RNA was extracted from OA-CHs using TRIzol reagent, followed by reverse transcription reaction and amplification reaction. Agarose-gel electrophoresis was implemented for semi-quantitative analysis of PCR products using the Gelpro32 gel image analysis software. Relative quantitative analysis was performed using 2^−ΔΔCt^ with β-actin as the internal reference. All used primers were as follows: lncRNA HOTAIR: the forward primer: 5'-CCATAGCCGATTAGCTGTCA' and the reverse primer: 5'-AATGCCGAACTGGAGGTG-3'; β-actin: the forward primer: 5'-GGCAAATGTCAGAGGGTTCT-3' and the reverse primer: 5'-TTCTTAAATTGGGCTGGGTC-3'.

### Enzyme-linked immunosorbent assay (ELISA)

The supernatant of OA-CHs was collected and centrifuged at 2500 r/min for 20 min, and the precipitation was discarded. The supernatant was added to the ELISA plate (50 µL per well) and incubated at 37 °C for 30 min. After the plate was sealed with a sealing plate membrane, the levels of TNF-α, IL-10, APN, and adiponectin receptor 2 (ADIPOR2) were detected using the ELISA method strictly in the light of the kit instructions.

### CCK-8 assay

The viability of OA-CHs was measured using CCK-8 assay kit (BIOSS, Beijing, China). At first, 3 × 10^4^ OA-CHs were seeded into each well of 96-well plates and cultured until reaching 70–90% confluence. Logarithmically growing cells were transfected by the above method. OA-CHs in 3 wells in each group were cultured for 0, 12, 24, 48, and 72 h, respectively. Next, 10 mL CCK-8 solution was supplemented into each well at each point of time, followed by incubation at 37 °C for 1–4 h. The viability of OA-CHs was determined by measuring the absorbance value of each well at 450 nm.

### Western blot analysis

The cells were lysed with radio-immunoprecipitation assay lysis solution and centrifuged at 12000 r/min for 10 min to harvest the total proteins, followed by sodium dodecyl sulfate–polyacrylamide gel electrophoresis (SDS-PAGE) (Beyotime, Shanghai, China). The 5 × SDS-PAGE protein loading buffer was added into cells at the ratio of 1:4 to obtain protein samples. The samples were heated for 10 min in a boiling water bath and supplemented into SDS-PAGE gel wells (5–10 µL per well). Filter papers and polyvinylidene fluoride membrane of the same size as the rubber strip were cut in advance (soaked in methanol for 2–3 min in advance) and immersed in the rotating film buffer for 5 min. Bubbles were removed at every step and the flow membrane was constant. After the protein was transferred into the membranes, the membranes were cooled to room temperature before the insertion of the membranes into the prepared Western washing solution and the removal of the membrane transfer solution by 5-min washing. The membranes were blocked with 5% skim milk powder at room temperature for 2 h and then washed with tris-buffered saline-tween (TBST) buffer 3 times (10 min/time). The membranes underwent overnight incubation with primary pig antibodies to phosphorylation (p)-AKT, PI3K, PTEN, AKT, P-PI3K, APN, ADIPOR1, and ADIPOR2 at 4 °C. The horseradish peroxidase-tagged secondary antibody was diluted with secondary antibody diluent at the ratio of 1:20,000 and used for incubation with the membranes at room temperature for 1 h. The ratio of the absorbance of target proteins to that of glyceraldehyde-3-phosphate dehydrogenase was calculated after electrogenerated chemiluminescence development, darkroom exposure, fixation, and photography.

### Molecular modeling

The two-dimensional structure diagram of active components of baicalin was downloaded from Traditional Chinese Medicine Systems Pharmacology (TCMSP) database. The compound structure and AKT1 were converted to PDBQT format by AutoDockTools-1.5.7 software, and the active pockets were formed. Finally, the PDBQT ligand obtained by entering CMD command and the PDBQT core protein obtained by AutoDockTools-1.5.7 were imported into Pymol for docking.

### Statistical analysis

SPSS statistical software 23.0 (IBM Corp. Armonk, NY, USA) was applied for statistical analysis, and GraphPad Prism software 8.2 (GraphPad Software, La Jolla, CA, USA) was utilized to capture images. The difference between groups was compared using Student's paired two-tail *t* test or Kruskal–Wallis nonparametric test. Classification variables were compared using chi-square test. Spearman correlation analysis was employed to evaluate the correlation of lncRNA HOTAIR with erythrocyte sedimentation rate (ESR), high sensitivity C-reactive protein (hs-CRP), immunoglobulin A (IgA), and other indicators. Data were expressed as mean ± standard deviation or median (quartile range). *p* < 0.05 was indicative of statistical significance.

## Results

### Clinical characteristics of OA patients and NC subjects

A total of 80 subjects were enrolled, including 30 NC subjects (10 males and 20 females, the age quartile was 55.50 (42.75, 70.0)) in the control group and 50 OA patients (17 males and 33 females, the age quartile was 55.0 (48.5, 65.25)) in the OA group. There were no statistically significant differences in age and gender distribution between the two groups, but there were significant differences in ESR, TC, TG, IgA, and C3 (Table 1).

**Table 1:** Changes in clinical lipid metabolism indexes and immune-inflammatory indexes in OA patients

Indexes	OA (*n *= 50)	NC (*n *= 30)	*p*
Gender (Female)	33^c^	20^c^	0.951
Age (years)	55.0 (48.5, 65.25)^a^	55.50 (42.75, 70.0)^a^	0.952
ESR (mm/h)	19.4 (11.2, 31)^a^	9.50 (5.66, 11.62)^a^	< 0.01
TC (mmol/L)	5.25 ± 1.29^b^	2.20 (1.53, 3.10)^a^	< 0.01
TG (mmol/L)	1.00 (1.35, 1.73)^a^	0.50 (0.34, 1.03)^a^	< 0.01
IgA (g/L)	2.48 ± 1.12^b^	1.61 (1.20, 2.20)^a^	< 0.01
C3 (g/L)	1.15 ± 0.22^b^	1.05 (0.94, 1.09)^a^	< 0.01
SDS score	53.09 ± 7.11^b^	NA	-
VAS score	5.72 ± 0.60^b^	-	*-*
SAS score	43.38 (40.62, 47.67)^a^	-	*-*

^a^Wilcoxon signed-rank test, median (25–75th percentile)

^b^mean ± standard error

^c^chi-square test

*NC* healthy control; *OA* osteoarthritis; *NA* not applicable; *ESR* erythrocyte sedimentation rate; *TC* total cholesterol; *TG* triglyceride; *IgA* immunoglobulin A; *C3* complement C3; *SDS* Self-rating Depression Scale; *SAS* Self-rating Anxiety Scale; *VAS* Visual Analog Scale

### LncRNA HOTAIR expression was upregulated in the PBMCs of OA patients

**Fig. 1:**
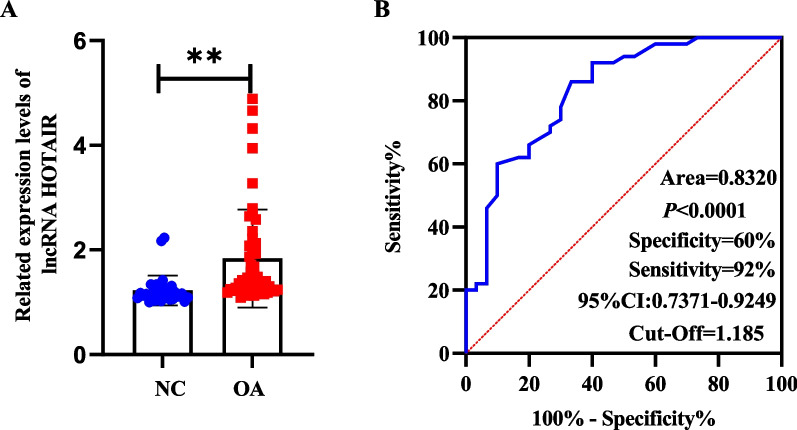
LncRNA HOTAIR was highly expressed in the PBMCs of OA patients. **A** LncRNA HOTAIR expression in OA patients was higher than that in NC subjects. **B** Comparison of ROC curves of lncRNA HOTAIR expression in PBMCs of OA patients and NC patients. **p < 0.01

This study intended to assess the lncRNA HOTAIR expression pattern in OA. LncRNA HOTAIR expression in the PBMCs of 50 OA patients and 30 NC subjects was detected using RT-qPCR. The findings revealed that lncRNA HOTAIR expression was increased in the PBMCs of OA patients (Fig. 1A). The diagnostic efficacy of lncRNA HOTAIR was evaluated using receiver-operating characteristic (ROC) curve analysis, and the area under the ROC curve (AUC) was 0.8310 [95% confidence interval (CI): 0.74–0.92]. According to the Youden index, the ideal cut-off value for identification of OA patients from NC subjects was 1.185 with a sensitivity of 60.00% and a specificity of 92.67% (Fig. 1B).

### Differential expression of inflammatory cytokines and APN in OA had a positive correlation with lncRNA HOTAIR expression

**Fig. 2:**
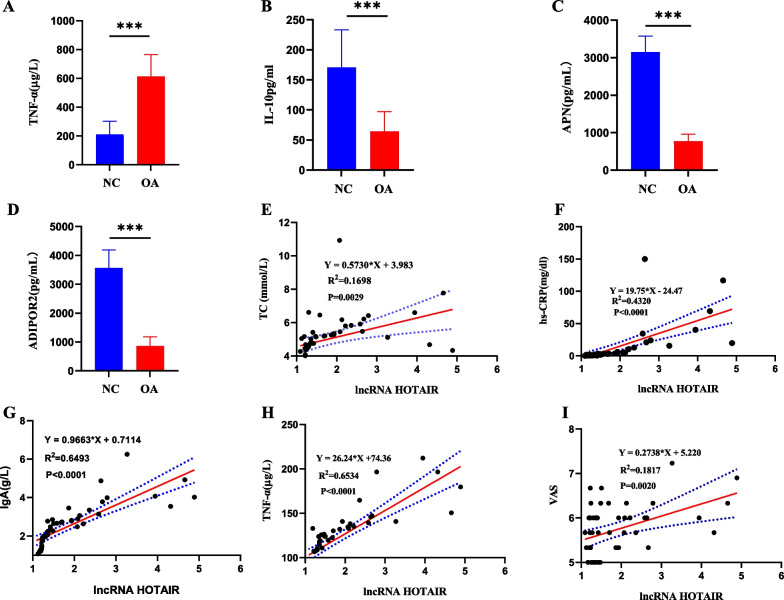
Differential expression of inflammatory cytokines in OA. **A**–**D** The levels of TNF-α (**A**), IL-10 (**B**), APN (**C**), and ADIPOR2 (**D**) in the serum of OA patients and normal subjects. ^***^ *p* < 0.001. (F-I) Correlation analyses of lncRNA HOTAIR with TC (**E**), hs-CRP (**F**), IgA (**G**), TNF-α (**H**), and VAS (**I**) in the PBMCs of OA patients

IL-10 was dramatically reduced (*p* < 0.01), while TNF-α was elevated (*p* < 0.01) in the OA group,, as compared to the control group, indicating the significant alterations in the levels of pro-inflammatory and anti-inflammatory factors in OA (Fig. 2A–B). Furthermore, the levels of APN and ADIPOR2 were considerably reduced in the OA group compared with those in the control group (*p* < 0.01; Fig. 2C–D). Then, we conducted correlation analyses of lncRNA HOTAIR with TC, hs-CRP, IgA, TNF-α, and VAS, respectively. The results displayed that lncRNA HOTAIR was a risk factor for TC, hs-CRP, IgA, TNF-α, and VAS [18] (Fig. 2E–I). These results further suggested the close relationship between lncRNA HOTAIR and the occurrence and development of OA.

### Association rule analysis of lncRNA HOTAIR with laboratory indexes in OA patients

Association rule analysis showed that the elevation of lncRNA HOTAIR was strongly correlated with the elevation of GSH, ESR, and VAS in OA patients. There was a strong correlation between the increase of lncRNA HOTAIR and the decrease of LDL-C, with a support degree greater than 20% and a confidence degree greater than 30%. The results are shown in Table 2.

**Table 2:** Association rule analysis between lncRNA HOTAIR and laboratory indicators in OA patients

Items (LHS ⇒ RHS)	Support (%)	Confidence (%)	Lift	*p* value
{lncRNA HOTAIR↑}⇒{Hospitalization days↓}	63.636	76.087	1.02	< 0.01
{lncRNA HOTAIR↑}⇒{sex (female)}	56.364	67.391	1.05	< 0.01
{lncRNA HOTAIR↑}⇒{SDS↑}	63.636	76.087	1.03	< 0.01
{lncRNA HOTAIR↑}⇒{ESR↑}	61.818	73.913	1.03	< 0.01
{lncRNA HOTAIR↑}⇒{VAS↑}	67.273	80.435	1.01	< 0.01
{lncRNA HOTAIR↑}⇒{LDL-C↑}	27.273	32.609	1.02	< 0.01

For correlations, Aprior module analysis was used. The minimal level of support was set at 20%, while the minimum level of confidence was set at 30%. When the degree of the lift was set to greater than 1, it was deemed important

### Optimal concentration of PBMCs, baicalin, and si-lncRNA HOTAIR, and effect of overexpression or knockdown of lncRNA HOTAIR on cell viability

**Fig. 3:**
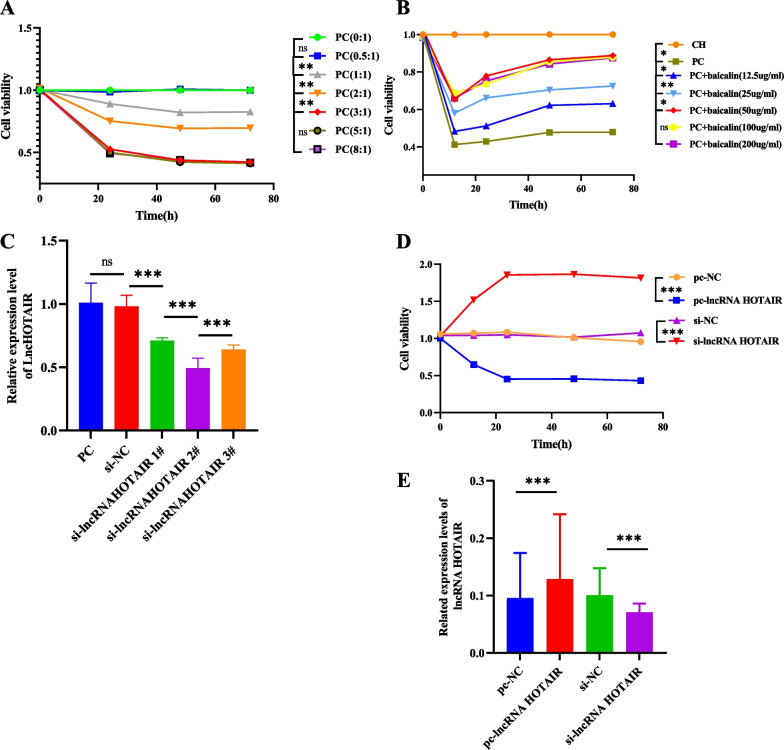
The optimal stimulation concentration of PBMCs (3:1, 48 h), baicalin (50 ug/ml, 48 h), and si-lncRNA HOTAIR 2# was screened and lncRNA HOTAIR silencing facilitated the viability of OA-CHs. **A** PBMCs: OA-CHs screening of the optimal concentration. **B** Baicalin screening of the optimal concentration. **C** Screening of si-lncRNA HOTAIR. **D** Effect of lncRNA HOTAIR on the viability of OA-CHs was tested by CCK-8 assay. **E** LncRNA HOTAIR expression in OA-CHs was measured by RT-qPCR. PC, PBMCs: OA-CHs. pc, pcDNA3.1. All tests were repeated three times and results were expressed as mean ± standard deviation. ^*^p < 0.05, ^**^p < 0.01, ^***^p < 0.001

OA-CHs were treated with different concentrations of OA-PBMCs and baicalin. The optimal stimulation concentration of PBMCs (3:1, 48 h), baicalin (50 ug/ml, 48 h), and si-lncRNA HOTAIR 2# was determined by CCK-8 assay (Fig. 3A–C) for subsequent experiments. The effect of lncRNA HOTAIR on the viability of OA-CHs was assessed by CCK-8 assay. The results exhibited that the PCDNA3.1-lncRNA HOTAIR group had dramatically boosted cell viability when compared to the PCDNA3.1-NC group. In contrast to the si-NC group, the cell viability of the si-lncRNA HOTAIR group was evidently diminished. Among the four groups, the viability of cells in the si-lncRNA HOTAIR group was the highest and that of cells in the lncRNA HOTAIR group was the lowest (Fig. 3D). LncRNA HOTAIR expression in the PCDNA3.1-lncRNA HOTAIR group was substantially higher (*p* < 0.01) than that in the PCDNA3.1-NC group, whereas lncRNA HOTAIR expression in the si-lncRNA HOTAIR group was significantly lower than that in the si-NC group (*p* < 0.01) (Fig. 3E).

### LncRNA HOTAIR overexpression facilitated TNF-α expression but repressed IL-10 expression in OA-CHs

**Fig. 4:**
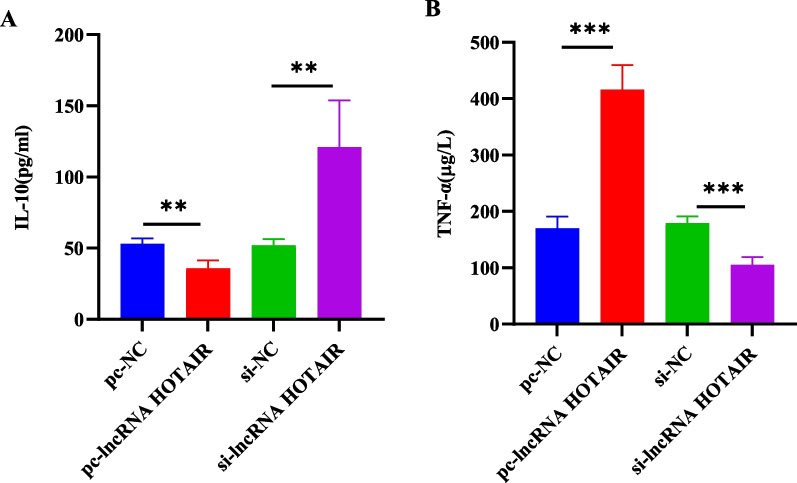
LncRNA HOTAIR overexpression caused TNF-α upregulation and IL-10 downregulation in OA-CHs. **A**–**B** The levels of IL-10 (**A**) and TNF-α (**B**) in OA-CHs were determined by ELISA. pc, pcDNA3.1. ^**^p < 0.01, ^***^p < 0.001

The levels of cytokines were evaluated by ELISA. Compared with that in the PCDNA3.1-NC group, TNF-α expression in the PCDNA3.1-lncRNA HOTAIR group was prominently augmented and IL-10 expression was remarkably decreased (*p* < 0.01). Compared with that in the si-NC group, TNF-α expression was markedly reduced and IL-10 expression was noticeably elevated in the si-lncRNA HOTAIR group (*p* < 0.01; Fig. 4A–B).

### LncRNA HOTAIR manipulated the PTEN/PI3K/AKT pathway in OA-CHs

**Fig. 5:**
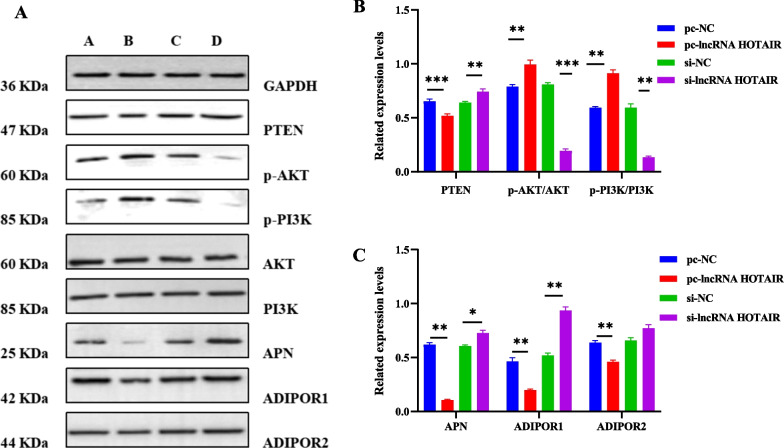
Effect of lncRNA HOTAIR overexpression or silencing on the PTEN/PI3K/AKT pathway in OA-CHs. **A** Representative Western blots of PI3K, p-AKT, p-PI3K, AKT, APN, ADIPOR1, ADIPOR2, and PTEN in OA-CHs. **B** Quantitative analysis of the expression of p-AKT/AKT, p-PI3K/PI3K, and PTEN in OA-CHs. **C** Quantitative analysis of the expression of APN, ADIPOR1, and ADIPOR2 in OA-CHs. pc, pcDNA3.1. All experiments were repeated three times and data were expressed as mean ± standard deviation. ^*^p < 0.05, ^**^p < 0.01, ^***^p < 0.001

To ascertain the impact of lncRNA HOTAIR on the PTEN/PI3K/AKT pathway, we adopted Western blot analysis to identify the expression of PI3K, p-AKT, p-PI3K, AKT, APN, ADIPOR1, ADIPOR2, and PTEN. As discovered in Fig. 5, overexpression of lncRNA HOTAIR enhanced the expression of p-PI3K and p-AKT and reduced the expression of APN, ADIPOR1, and PTEN. On the contrary, lncRNA HOTAIR silencing contributed to the opposite trends. However, the overexpression or silence of lncRNA HOTAIR did not have a significant effect on the expression of PI3K, AKT, and ADIPOR2.

### Molecular docking between baicalin and AKT1

**Fig. 6:**
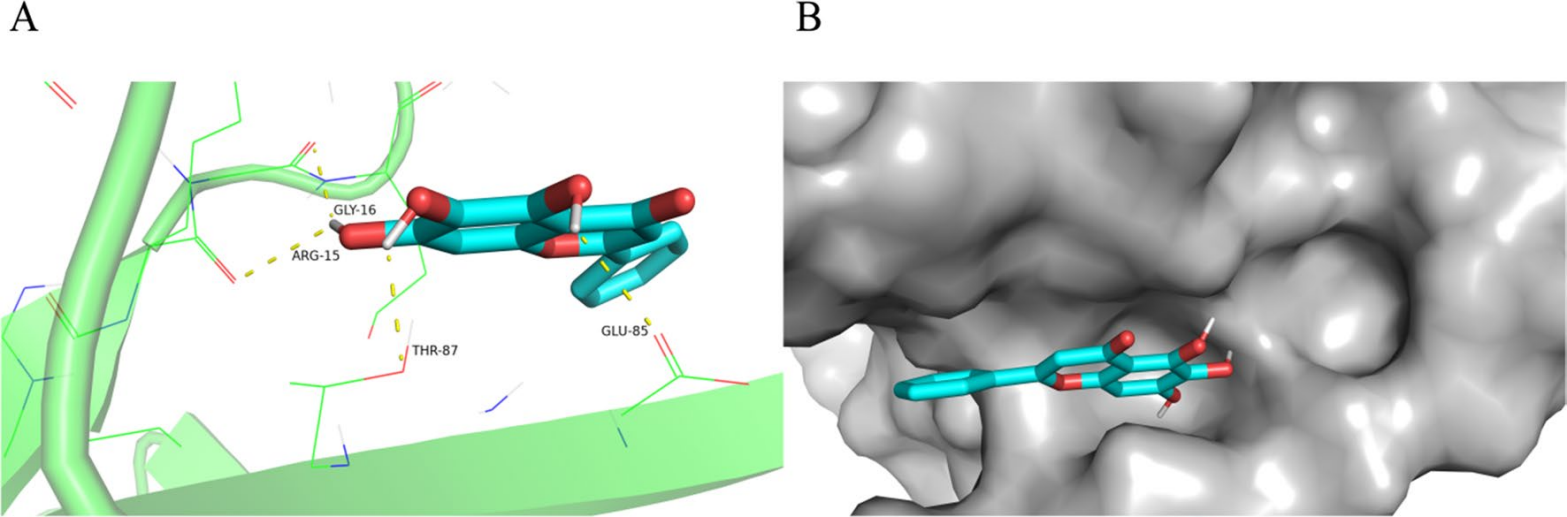
Molecular Docking between Baicalin and AKT1. **A** Macro- and local-level views between the residues of ARG-15, THR-87, GLY-16, and GLU-85 in baicalin and AKT1. **B** Space-filling model

To explore the mechanism of baicalin, we next performed computational molecular docking analysis to evaluate whether there was any affinity between baicalin and RAC-alpha serine/threonine-protein kinase (AKT1) protein. The chemical structure of baicalin was employed, and it was discovered that baicalin interacted with and docked at the AKT1 binding site (Fig. 6A). The space-filling model was also used to illustrate the interaction between baicalin and AKT1 (Fig. 6B). High-affinity (− 6.06 kcal/mol) hydrogen binding events were observed between the residues of ARG-15, THR-87, GLY-16, and GLU-85 in baicalin and AKT1. These results indicated that baicalin probably inhibited the development of OA by interacting with the AKT1.

### Baicalin and si-lncRNA HOTAIR exerted synergistic effects on the PTEN/PI3K/AKT signaling pathway, APN, and inflammatory factors

**Fig. 7:**
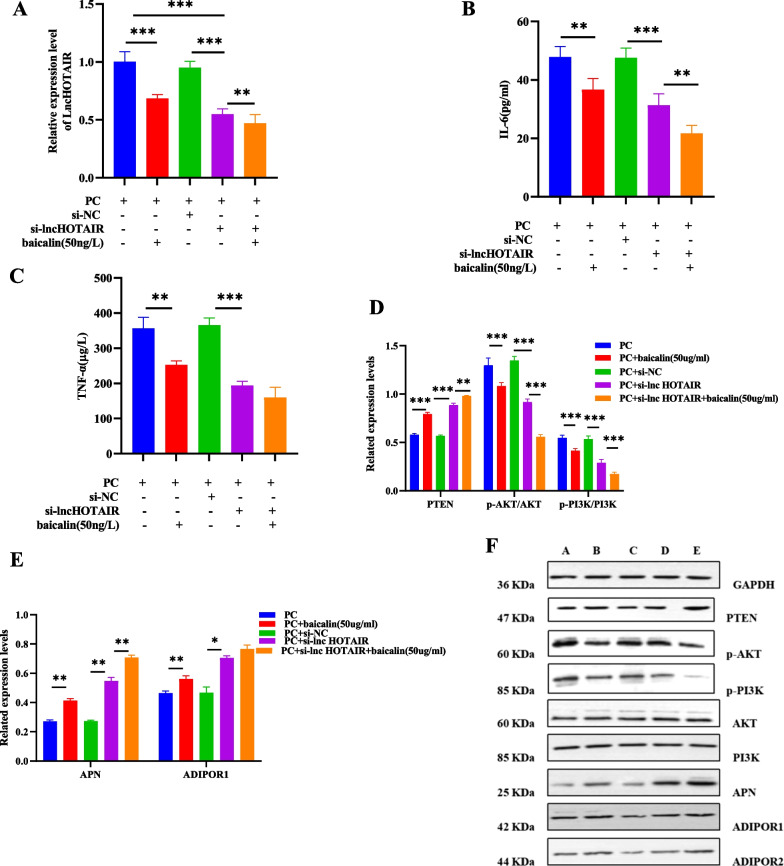
Baicalin and si-lncRNA HOTAIR had synergistic effects on the PTEN/PI3K/AKT signaling pathway, APN, and inflammatory factors. After lncRNA HOTAIR knockdown and/or baicalin intervention, the protein expression of lncRNA HOTAIR, IL-6, and TNF-α was evaluated by RT-qPCR and ELISA (**A**–**C**). The protein expression of p-AKT/AKT, p-PI3K/PI3K, PTEN, APN, and ADIPOR1 was evaluated by Western blot assay (**D**–**F**). PC, PBMCs: OA-CHs. All results were represented as the mean ± standard deviation. ^*^p < 0.05, ^**^p < 0.01, ^***^p < 0.001; *n* = 3

To evaluate the contribution of baicalin relieving OA, we explored the effect of baicalin on the PTEN/PI3K/AKT signaling pathway, APN, and inflammatory factors after si-lncRNA HOTAIR treatment in OA-PBMCs + OA-CHs. The results showed that baicalin or si-lncRNA HOTAIR inhibited the expression of lncRNA HOTAIR and reduced the levels of IL-6 and TNF-α (Fig. 7A–C). In addition, baicalin reduced the levels of p-AKT and p-PI3K proteins and increased the levels of PTEN, APN, and ADIPOR1 proteins, showing a similar effect with si-lncRNA HOTAIR. Besides, there was no synergistic effect in reducing IL-6 and increasing APN. For the rest of indicator, baicalin and si-lncRNA HOTAIR had a certain degree of synergy.

## Discussion

As the most prevalent chronic joint disease, OA causes cartilage loss, joint pain, and stiffness [19, 20]. Aging and obesity are well-known risk factors for OA, leading to a high susceptibility to joint injuries [21]. The critical role of inflammation in the pathophysiology of OA has been well-documented. Synovial inflammation resulting in the changes in the levels of inflammatory factors such as IL-10 and TNF-α has been associated with the radiographic and pain progression of OA [22–24]. TNF-α as an essential pro-inflammatory factor can induce the activation of fibroblast-like synoviocytes in OA and cause the gradual destruction of cartilage. IL-10 is known to be a potent anti-inflammatory cytokine, and IL-10 deregulation plays a role in the development of OA. Moreover, existing studies [25, 26] have shown that lipid metabolism imbalance contributes to the occurrence and development of OA, and hyperlipidemia is closely related to the immune-inflammatory response of OA patients. APN, an indicator related to lipid metabolism, induces inflammatory responses [27]. Kabalyk et al. [28] have confirmed that hyperlipidemia directly acts on articular tissues and causes cellular stress, manifested as changes in morphological and functional characteristics of CHs. Processes including cell death, pathological mineralization of articular cartilages, and enhanced pathological angiogenesis can be observed in hyperlipidemia. The quantity of inflammatory cytokines (including IL-1β, IL-6, IL-8, and TNF-α) is significantly augmented in a lipopolysaccharide-induced C28/I2 human chondrocyte cell model compared with the corresponding control group [29, 30].

LncRNA research constitutes a novel and promising field in understanding the complexity of OA pathogenesis. The involvement of lncRNA HOTAIR in OA progression has been reported by several previous studies [31–33]. For example, it is reported that lncRNA HOTAIR is highly expressed in OA, and participates in IL-1β-induced matrix metalloproteinase overexpression and promotes inflammatory responses in CHs [34]. A prior research has also elucidated that the PI3K/AKT pathway assumes a key role in the development of OA inflammation [35]. Moreover, the PI3K/AKT pathway exerts a certain effect on lipid metabolism [36].

This study provided evidence of substantially high expression of lncRNA HOTAIR in OA-PBMCs and OA-CHs with the AUC of 0.8310, the optimal truncation value of 1.185, the sensitivity of 60.00%, and the specificity of 92.67%, illustrating that lncRNA HOTAIR had the high diagnostic value. Our results also exhibited that IL-10, APN, and ADIPOR2 levels were diminished and TNF-α level was augmented in OA patients. APN can control inflammation, functioning as a potential therapeutic target for OA [37]. All changes in the levels of cytokines, APN, and its receptors can be indicated as the deregulation of pro-inflammatory and anti-inflammatory factors. In addition, clinical trials have depicted that lncRNA HOTAIR shares a positive correlation with TNF-α, clinical immune-inflammatory indicators, lipid metabolism indexes, and patient perception score scales, like TC, hs-CRP, IgA, and VAS. LncRNA HOTAIR dysregulation is partially responsible for the imbalance of inflammatory factors. To further dissect out the effects of lncRNA HOTAIR on the proliferation, inflammation, and lipid metabolism of OA-CHs, we conducted in vitro cell experiments by stimulating OA-CHs with OA-PBMCs.

Subsequently, the impact of lncRNA HOTAIR on the function of OA-CHs was assessed using CCK-8 assay. The results showed that the viability of OA-CHs was conspicuously decreased after overexpressing lncRNA HOTAIR. On the contrary, the cell viability was enhanced following the transfection of lncRNA HOTAIR silencing plasmids. Under the condition of lncRNA HOTAIR overexpression and silencing, inflammatory cytokines were further measured using ELISA method. The results displayed that overexpression of lncRNA HOTAIR significantly enhanced TNF-α level and reduced IL-10 level. In contrast, the deletion of lncRNA HOTAIR triggered an opposite tendency to inflammation. SiRNAs, as small non-coding RNA fragments, are one of RNA interference inducers for gene modulations, and the activity of inflammatory responses can be affected by using specific siRNAs to regulate the expression of particular inflammatory cytokines [38, 39]. These results suggested that si-lncRNA HOTAIR might be implicated in the occurrence and development of OA by orchestrating the levels of inflammatory factors. Consistently, lncRNA HOTAIR silencing has also been demonstrated to restrain cell viability via the PI3K/AKT pathway, reduce the cellular inflammatory response, inhibit drug resistance, and improve the quality of life of patients [40–43].

The effect of lncRNA HOTAIR on inflammatory factors may be related to its regulation of APN and its receptors, as well as the inflammatory pathway PTEN/PI3K/AKT. The current study noted the potential correlation between lncRNA HOTAIR and PTEN/PI3K/AKT pathway in OA-CHs through cell experiments. Western blot analysis was implemented to determine the influence of lncRNA HOTAIR on the pathway-related proteins, APN, and its receptors. The results manifested that overexpression of lncRNA HOTAIR elevated the expression of p-PI3K and p-AKT but reduced the expression of PTEN, APN, and ADIPOR1 proteins. The reason why overexpression of lncRNA HOTAIR had no significant effect on the expression of ADIPOR2 may be that ADIPOR1 is expressed abundantly in cartilages, bones, and synovial tissues, whereas ADIPOR2 is rarely detected [44]. Notably, baicalin as a natural flavonoid glycoside with significant anti-inflammation activity can inhibit the PI3K/AKT signaling pathway to induce the apoptosis and autophagy of human osteosarcoma cells [45]. Similarly, our subsequent cell experiments also confirmed that baicalin and lncRNA HOTAIR silencing exerted synergistic effects on the PTEN/PI3K/AKT signaling pathway inflammatory factors.

In summary, our obtained findings uncovered that lncRNA HOTAIR was highly expressed in the PBMCs of OA patients. LncRNA HOTAIR modulated the viability of OA-CHs and participated in inflammatory responses of OA by influencing the levels of inflammatory cytokines, APN, and the PTEN/PI3K/AKT pathway in OA-CHs, thus afflicting the occurrence and development of OA. These findings suggest the potential of lncRNA HOTAIR as a biomarker for OA treatment and provide novel insights into the pathogenesis of OA. Molecular docking analysis revealed that baicalin and si-lncRNA HOTAIR had synergistic effects on the PTEN/PI3K/AKT signaling pathway, APN, and inflammatory factors. However, whether baicalin alleviates immune inflammatory responses in OA patients by interfering the expression of lncRNA HOTAIR or the activation of the PTEN/PI3K/AKT signaling pathway remains to be further verified.
